# Objective wearable measures and subjective questionnaires for predicting response to neurostimulation in people with chronic pain

**DOI:** 10.1186/s42234-023-00115-4

**Published:** 2023-06-21

**Authors:** Robert Heros, Denis Patterson, Frank Huygen, Ioannis Skaribas, David Schultz, Derron Wilson, Michael Fishman, Steven Falowski, Gregory Moore, Jan Willem Kallewaard, Soroush Dehghan, Anahita Kyani, Misagh Mansouri

**Affiliations:** 1 Spinal Diagnostics, Tualatin, OR USA; 2 Nevada Advanced Pain Specialists, Reno, NV USA; 3 Erasmus University Medical Center, Rotterdam, Netherlands; 4 Expert Pain, Houston, TX USA; 5 Nura Clinics, Minneapolis, MN USA; 6 Goodman Campbell Brain & Spine, Carmel, IN USA; 7 Center for Interventional Pain and Spine, Lancaster, PA USA; 8 Neurosurgical Associates of Lancaster, Lancaster, PA USA; 9 Pacific Sports and Spine, Eugene, OR USA; 10 Rijnstate Hospital, Arnhem, Netherlands; 11 Abbott, Austin, TX USA

**Keywords:** Chronic pain therapy, Spinal cord stimulation, Patient-reported outcomes, Wearable technology, Digital biomarkers for pain, Dimensionality reduction, Questionnaire burden, Machine learning, Predictive modeling, PGIC

## Abstract

**Background:**

Neurostimulation is an effective therapy for treating and management of refractory chronic pain. However, the complex nature of pain and infrequent in-clinic visits, determining subject’s long-term response to the therapy remains difficult. Frequent measurement of pain in this population can help with early diagnosis, disease progression monitoring, and evaluating long-term therapeutic efficacy. This paper compares the utilization of the common subjective patient-reported outcomes with objective measures captured through a wearable device for predicting the response to neurostimulation therapy.

**Method:**

Data is from the ongoing international prospective post-market REALITY clinical study, which collects long-term patient-reported outcomes from 557 subjects implanted by Spinal Cord Stimulator (SCS) or Dorsal Root Ganglia (DRG) neurostimulators. The REALITY sub-study was designed for collecting additional wearables data on a subset of 20 participants implanted with SCS devices for up to six months post implantation. We first implemented a combination of dimensionality reduction algorithms and correlation analyses to explore the mathematical relationships between objective wearable data and subjective patient-reported outcomes. We then developed machine learning models to predict therapy outcome based on the subject’s response to the numerical rating scale (NRS) or patient global impression of change (PGIC).

**Results:**

Principal component analysis showed that psychological aspects of pain were associated with heart rate variability, while movement-related measures were strongly associated with patient-reported outcomes related to physical function and social role participation. Our machine learning models using objective wearable data predicted PGIC and NRS outcomes with high accuracy without subjective data. The prediction accuracy was higher for PGIC compared with the NRS using subjective-only measures primarily driven by the patient satisfaction feature. Similarly, the PGIC questions reflect an overall change since the study onset and could be a better predictor of long-term neurostimulation therapy outcome.

**Conclusions:**

The significance of this study is to introduce a novel use of wearable data collected from a subset of patients to capture multi-dimensional aspects of pain and compare the prediction power with the subjective data from a larger data set. The discovery of pain digital biomarkers could result in a better understanding of the patient’s response to therapy and their general well-being.

**Supplementary Information:**

The online version contains supplementary material available at 10.1186/s42234-023-00115-4.

## Background

Chronic pain is a debilitating condition that affects patients' quality of life by limiting social interaction, physical activity, work participation, and sleep quality as well as increasing the risk of fatigue, anxiety, and depression (Cohen et al. 2021; Crofford 2015). It is estimated that more than 50.2 million (or one in five) adults in the US experience chronic pain (Steingrímsdóttir et al. 2017). The social and individual burdens associated with this condition have escalated worldwide. Treatment options for chronic pain are varied, depending not only on the underlying etiology of pain, but also on factors such as insurance access, geographic location, and the type of physician a patient may see. Traditional treatment options range from conservative options such as patient education and reassurance, physical therapy, and chiropractic care, to intermediate and minimally invasive options such as pharmacologic agents, steroid injections, ablation, and neurostimulation), and to invasive options such as spine surgery.

Spinal Cord Stimulation (SCS) is a recommended intervention for the management of refractory chronic pain syndromes (Deer et al. n.d; Taylor et al. 2006; Vos et al. 2014; Stelter et al. 2021; Kumar et al. 2007). One's response to SCS therapy may differ from another's over time and maintaining optimal response to the therapy often demands interactive adjustment of treatment parameters; thus, appropriate patient selection and long-term monitoring of symptoms are crucial in optimizing the outcomes of SCS therapy (Goudman et al. 2022; Goudman et al. 2021a).

Accurate pain measurement in long-term follow up facilitates early diagnosis, disease progression monitoring, and therapeutic efficacy evaluation; thus, it is a vital component in managing chronic pain conditions. While clinical trials have typically relied on unidimensional metrics such as the Numerical Rating Scale (NRS) and Visual Analog Scale (VAS), the complexity of chronic pain demands the use of more robust, multidimensional tools to better measure pain and assess outcomes (Dworkin et al. 2005; Levy et al. 2023). More comprehensive patient-reported outcome measures (PROMs) include the PROMIS-29 (Cella et al. 2019; Hays et al. 2018), Pain Catastrophizing Scale (PCS) (Osman et al. 2000), Oswestry Disability Index (ODI) (Fairbank and Pynsent 2000), and Patient Global Impression of Change (PGIC) (Salaffi et al. 2004); these metrics are now routinely used to provide deeper insight into pain and the different areas that pain can impact function, mental health, and quality of life (Katz et al. 2021).

Although collecting of PROMs in person during a clinic visit is usually considered the gold standard in assessing pain (Dworkin et al. 2005), frequent collection of these PROMs is cumbersome for both patients and clinicians. In addition, the infrequency of clinic visits can make it difficult to track pain progression and fluctuations over time desirable for optimal treatment of chronic pain. There is also a chance of bias that impacts both the accurate assessment and treatment of chronic pain (Hoffman et al. 2016; Anderson et al. 2009). One potential solution to these challenges is the use of remote monitoring technologies, such as mobile apps and wearable devices, to collect PROMs and other relevant data from patients in real time (Rejula et al. 2021; Pathak et al. 2021). This approach has the potential to provide more frequent and objective assessments of pain, as well as other related factors such as physical activity and sleep quality, which can inform treatment decisions and help identify early warning signs of pain exacerbation.

Advances in wearable technology have enabled the measurement of “digital biomarkers” including movement and physical activity (Caramia et al. 2018; Maceira-Elvira et al. 2019; Yan et al. 2017; Motl et al. 2009; Smuck et al. 2017), gait and posture (Caramia et al. 2018; Maceira-Elvira et al. 2019; Yan et al. 2017; Motl et al. 2009; Smuck et al. 2017), neuromuscular and physiological signals (Kushioka et al. 2022), sleep (Goudman et al. 2021a; Pathak et al. 2021; Rodríguez-Fernández et al. 2021; Avila et al. 2021; Xia et al. 2019), and behavioral data (Chen et al. 2021; Naeini et al. 2019). Such markers are best collected in real time, outside of the physical confines of a medical office setting, and the use of wearable devices now makes this possible.

Despite recent research highlighting the importance of using machine learning techniques in pain research, previous works have focused on correlating and monitoring symptoms and side effects of pain with digital biomarkers, and not necessarily predicting the subject’s reported outcomes including response to SCS therapy (Smuck et al. 2017; Avila et al. 2021; Koenig et al. 2016a; Tomkins-Lane et al. 2022; Costa et al. 2022).

In this paper, we first utilized dimensionality reduction algorithm to evaluate the similarities between data obtained objectively through wearables and phone applications and subjective patient-reported outcomes. We then applied machine learning techniques to predict SCS therapy outcome evaluated by subject’s response to NRS or PGIC using both subjective questionnaires and objective measures using wearables. We hypothesized that many physical and psychological aspects of chronic pain or its effects on patients can be objectively measured and utilized to accurately predict SCS therapy outcomes.

## Method

Data for our analysis were extracted from the ongoing prospective, multicenter, international REALITY (Long-Term Real-World Outcomes Study on Patients Implanted with a Neurostimulator) study (NCT03876054). Before starting the study, Institutional Review Board or Ethics Committee approval was received at each site, and all patients were given written informed consent. The devices used in this study are FDA-approved or approved by a corresponding national agency for this indication. Study visits occur at enrollment, at baseline, peri-operatively, and at six months, one year, and yearly thereafter until the subject has been followed for five years post-implantation.

The eligibility criteria included a baseline pain score ≥ 6 on the 0–10 NRS and scheduled for implantation of an Abbott SCS or dorsal root ganglion (DRG) stimulation neurostimulation system (Abbott Neuromodulation, Austin, Texas) within 60 days of the baseline visit. To replicate the range of complex patients seen in daily medical practice, the REALITY study inclusion criteria was developed with few restrictions on the pain indication as permitted by regulatory guidelines in each geographical area and according to standard clinical practice.

Demographics, pain etiology, and chronic pain history were collected at baseline. Various patient-reported outcome measures, as described in detail below, were collected at baseline and each follow-up study visit in accordance with the IMMPACT recommendations (Dworkin et al. 2005) to capture the effects of therapy on subjects’ pain, function, disability, and mental health. Pain intensity was measured using NRS, where 0 is no pain and 10 is the worst pain imaginable (Farrar et al. 2001). Physical function and pain related disability were measured with the 10-item Oswestry Disability Index (ODI) (Fairbank and Pynsent 2000). Each section in the scale covers a different domain (pain intensity, personal care, lifting, walking, sitting, standing, sleeping, sex life, social life, and traveling). Emotional distress was assessed with the 13-item PCS which yields a total score and three subscale scores assessing rumination, magnification, and helplessness (Sullivan 1995). PROMIS-29 was used to assess the following nine domains: Physical Function and Ability to Participate in Social Roles and Activities, as well as the seven days average of subject’s Depression, Anxiety, Fatigue, Sleep Disturbance, Pain Interference, and Pain Intensity (Cella et al. 2010; Cella et al. 2007). Other standardized metrics such PGIC (Geisser et al. 2010), a 7-question scale to assess patient global health and a subject’s impression of clinical change, were also collected at each follow-up. Subjects were also asked to report their satisfaction with pain relief provided by the therapy on a five-scale rating of very satisfied, satisfied, neither satisfied nor dissatisfied, dissatisfied, very dissatisfied.

### REALITY Wearable Sub Study

The REALITY wearable sub-study was devised to investigate the feasibility of using smartwatch recordings to measure physiological and behavioral markers and to characterize patient experience from baseline to 6 months post-implantation in a subgroup of patients with access to a smartwatch. The sub-study visits occurred at enrollment, baseline, and at three- and six-months post-implantation. All sub-study participants were given an Apple® Watch (Series 3) at enrollment and were instructed to enter NRS scores on a custom watch application daily from baseline to six months after implantation. In addition to the NRS scores, the watch application passively collected several HealthKit metrics for activity, behavior, and cardiac measures, such as step count, stand time, distance walked/run, heart rate, and heart rate variability. Participants were asked to start the watch application at least once a day and the NRS data was sent to secure cloud storage after they selected their current pain level from 0 (no pain) to 10 (the worst pain imaginable). The REALITY iOS-based custom application was provided on the given iPhones to participants to collect behavioral data, PROMs, such as PROMIS-29, ODI, PCS, and PHQ-9 on a regular basis (at least 3 times pre-implant, and monthly post-implant). In addition, PGIC was collected at least monthly post-implant. Many subjects reported PGIC multiple times a month. The REALITY iPhone custom application is a companion to the watch application and when installed on the iPhone, it automatically initiates the installation of the watch application on the paired Apple® Watch.

### Data preprocessing and missing data

The statistical features of the daily windowed data were included in the feature set, such as maximum, minimum, sum, mean, standard deviation, and 25, 75, and 90 percentiles of data. To balance weights and missing data for low-resolution wearable features in our analyses, we used daily window averaging for data points with the same pain level. However, due to a high number of missing and low-resolution sleep data acquired through the smartwatch HealthKit app Series 3, this measure was excluded from further analysis. In addition, heart rate variability (HRV) calculated through the Apple® HealthKit had missing data points and inter-beat interval was not accessible, and therefore heart rate values were used to estimate the time interval for calculating HRV using three different methods: root mean square of successive inter-beat intervals of heartbeat differences (RMSSD), standard deviation of the average inter-beat intervals without artifacts (NN intervals) for each 5 min-period over a 24-h recording of HRV (SDANN), and the average of standard deviations of all the NN intervals for each 5 min-period over a 24-h recording of HRV (SDNNI) (Shaffer and Ginsberg 2017). To handle missing data for the subjective data from PROs, we used the average score across all data points within the same NRS and PGIC level depends on the output of the machine learning model meaning PGIC was used to do the imputation of missing PROs for NRS modeling and vice versa NRS for PGIC modeling.

### Dimensionality reduction

Principal component analysis (PCA) was used to understand the similarities across many subjective measures in both REALITY main study and sub study. The same analysis was performed to compare the subjective and wearable objective measures (WOMs) collected for all REALITY sub-study patients. PCA is a statistical method used for large datasets to reduce the dimensionality of the data while increasing the interpretability with minimal data loss (Abdi and Williams 2010). PROMs from all scales and WOMs were treated as unique entries. Data points from various scales were standardized using the z-score (standard score) prior to this analysis. The z-score describes the fractional distance between a data point and the population mean in terms of standard deviation units. Similarities between the clusters of PROMs and WOMs were compared.

### Predictive models using machine learning

Machine learning models were developed from baseline demographic and medical history, wearable objective measurements, and subjective PROMs collected as described previously. A balanced number of training sets for each class of different output variables was considered. Subject response to SCS therapy was evaluated based on how various objective and subjective input variables to the machine learning model can predict pain and PGIC categories. For prediction of PGIC, scores of PGIC were categorized into three responder groups; 1) subjects who selected “No change (or the condition has gotten worse)”, “Almost the same, or hardly any change at all”, “A little better, but no noticeable change”, and “Somewhat better, but the change has not made a real difference” were considered non-responders, 2) subjects who selected “Moderately better, and a slight but noticeable change”, “Better and a definite improvement that has made a real and worthwhile difference”, were considered moderate responders, and 3) subjects who selected “A great deal better and a considerable improvement that has made all the difference” were considered super responders. Similarly, for prediction of pain, NRS scores were categorized into three responder groups: mild (NRS < 4), moderate (NRS ≥ 4 and NRS ≤ 6), and severe (NRS > 6).

For the sub-study, Random Forest (RF) model (Breiman 2001) was implemented to predict PGIC and NRS levels using the PROMs and WOMs collected from the Apple® Watch. 80% of the data was used for training the model and model was tested on the remaining 20%. To increase the robustness of the predictions among the training sets, the Random Forest model was trained 50 times using randomly selected 80% of the input data available. The reported outcomes were then averaged across all 50 different runs. Accuracies of predictive models developed on both main study and sub-study with and without the objective measures were compared. The models were tested with different input variables such as PROMs models for REALITY main study meaning PROs were used as inputs to the random forest model to predict NRS and PGIC, and PROMs or WOMs models for REALITY wearable sub-study meaning PROMs and objective measures were used as inputs to predict NRS and PGIC. Machine learning models were developed using Scikit-lean library and hyper parameter tuning was performed to obtain the highest accuracy for each model.

## Results

At the time of data collection, 557 patients, 324 females, and 233 males, at 53 investigational sites (28 in the United States, 23 sites in the European Union, and two sites in Australia) had completed at least one follow-up visit. Table 1 shows the baseline demographic and medical history for all the subjects included in the analysis from the REALITY main study. Of all subjects implanted, 73.8% had SCS and 26.2% had DRG neurostimulators. A total of 1100 follow-up visits were included for the analysis in this paper for the main study; These included patient visits that collected and reported both NRS and PGIC at each visit. These include 336 visits at 6 months, 411 visits at 1 year, 251 visits at 2 years, and 102 visits at 3 years.

**Table 1:** Baseline demographics and medical history for all the subjects included in the analysis

		**Count**	**Mean**	**Standard Deviation**	**N (%)**
Age at Time of Consent		557	60.0	(13.8)	
Height (cm)		557	169.1	(10.3)	
Weight (kg)		557	88.4	(22.4)	
Systolic Blood Pressure		557	132.0	(18.6)	
Diastolic Blood Pressure		557	78.3	(10.8)	
Pain History (years)		557	10.2	(11.2)	
Sex at Birth	F				58.2%
	M				41.8%
Pain Etiology	Back/lower Limb PSPS^a^ type I				16.0%
	Back/lower Limb PSPS^a^ type II				32.3%
	Radiculopathy				9.3%
	CRPS^b^- I				15.1%
	CRPS^b^- II (Causalgia)				12.2%
	Chronic Post-surgical Pain				2.5%
	Peripheral Neuropathy^c^				5.9%
	Other Chronic Pain^d^				6.6%
Neurostimulation Therapy	SCS DRG				73.8% 26.2%
Current Work Status	Disabled	83			14.9%
	Not Working	329			59.1%
	Part Time	40			7.2%
	Full Time	105			18.9%
What impact pain has on subj life?	None	1			0.2%
	Very Mild	2			0.4%
	Mild	13			2.3%
	Moderate	114			20.5%
	Severe	266			47.8%
	Very Severe	161			28.9%
Frequency of subject’s exercise	None	297			53.3%
	< 1 Day/Week	37			6.6%
	1–2 Days/Week	76			13.6%
	3–5 Days/Week	94			16.9%
	> 5 Days/Week	53			9.5%

^a^*PSPS* Persistent Spinal Pain Syndrome; Type I: Non-surgical, Type II: Surgical

^b^*CRPS* Complex Regional Pain Syndrome

^c^(Poly)neuropathies including painful diabetic polyneuropathy and post herpetic neuralgia

^d^Other Chronic Pain” include Visceral Pain, Post-Amputation, and Neck and upper limb pain

Twenty participants (15 males and 5 females) were enrolled as part of the REALITY wearable sub-study. Of those, one participant withdrew consent prior to the permanent system implantation; All subjects in the sub-study were implanted with an SCS system. Two participants withdrew consent after permanent implantation; one participant’s participation was terminated by the investigator; one participant was excluded from analysis due to a lack of wearable data. The average and standard deviation of participant’s age was 52.25 (± 9.7) years and on average sub-study subjects experienced 12.3 (± 11.7) years of chronic pain at baseline. At baseline, 60% of subjects did not exercise, 15% exercised 1–2 days a week, 15% exercised 3–5 days a week, and 10% exercised greater than 5 days a week. Similarly, 25% of subjects were employed full-time, 15% part-time, 40% not employed, and 20% disabled. The impact of pain on the subject life was severe or very severe in 65% of subjects, moderate in 30%, and mild in 5% of subjects. Back pain was the primary pain diagnosis of the majority of the participants (85%) in the sub-study cohort; 40% of subjects suffered from back/lower limb PSPS type II, 20% from back/lower limb PSPS type I, 25% from radiculopathy, and15% from CRPS Type I. Each study visit was treated as a separate data point in the analysis regardless of the subject identification. Over the course of the 6-month sub study, all 15 subjects provided a total of 142 data points consisting of both subjective and objective data, which were then included in the analysis.

### Wearable data compliance

The compliance rates for both completing PROMs on the iPhone and wearing the smart watch were relatively high, with median compliance rates of 88.8% and 84.7%, respectively for the sub-study participants. Participants were considered compliant if they completed the PROs at least three times during the baseline and once every month after the implant for a duration of 6 months, or if they wore the smart watch for at least 7 days during the baseline and 180 non-consecutive days after the implant. There was no significant difference in compliance rates between completing PROs on the iPhone and wearing the smart watch, as determined by a Wilcoxon rank-sum test. To ensure compliance, participants who missed providing data for more than 3 consecutive days or completing PROMs on the custom application received follow-up phone calls from clinic staff.

### PCA results

**Fig. 1:**
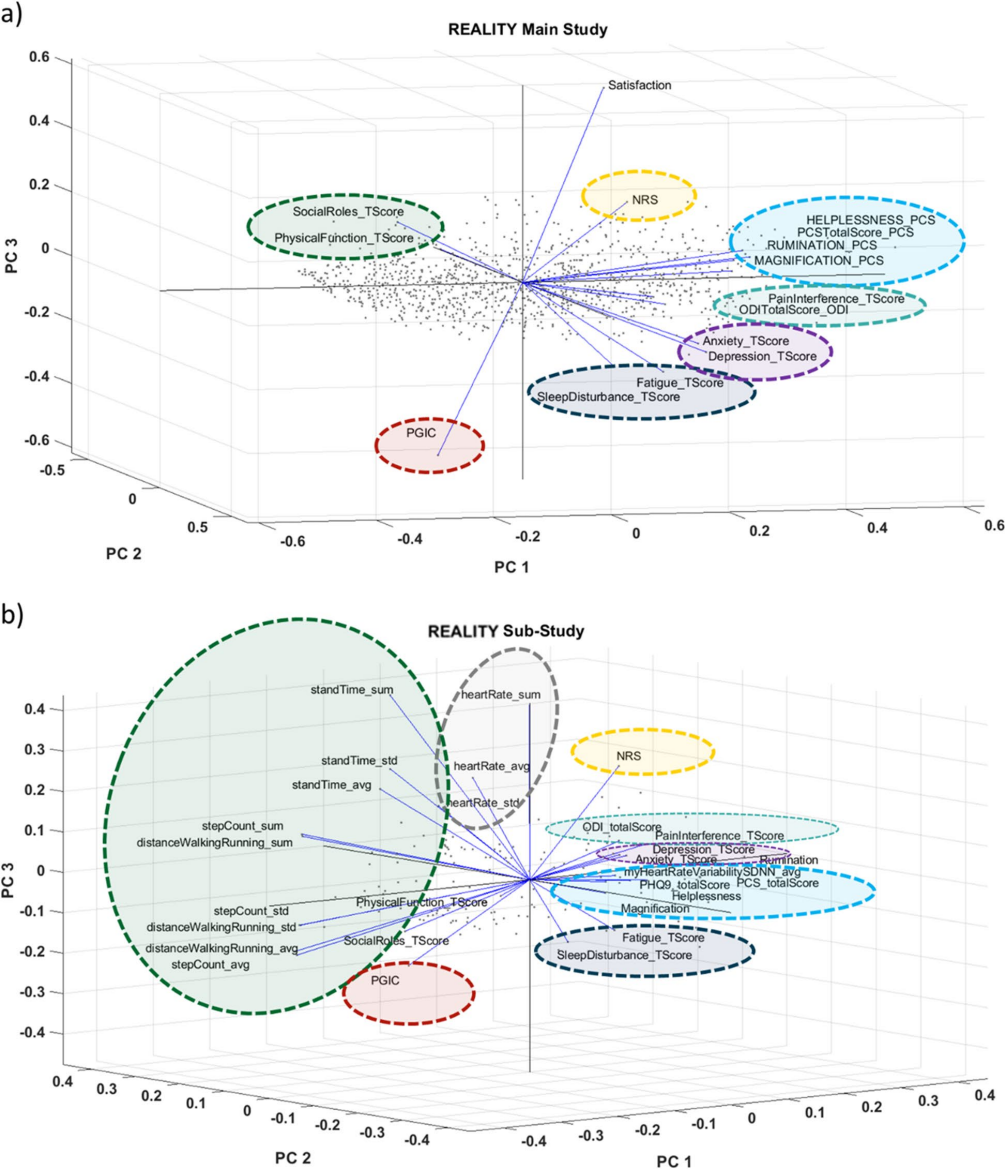
PCA loadings of top three principal components in a) the REALITY main study, and b) the REALITY sub-study

**Fig. 2:**
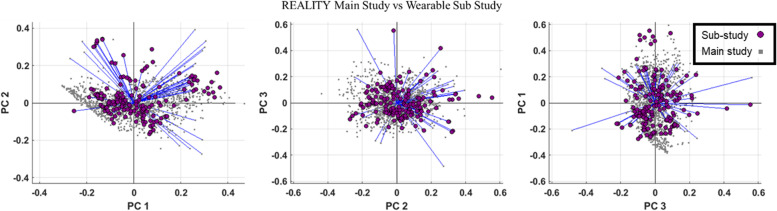
Comparison of scores (samples) for the first 3 principal components between REALITY main study (gray dots) and wearable sub-study (purple dots)

Figure 1a and b show the PCA results on all PROMs in the main study and the PROMs and WOMs in the wearable sub-study, respectively. In both main study and sub-study, psychological aspects of pain characterized by the PCS total score, PCS helplessness, PCS magnification, and PCS rumination were grouped together. Similarly, in both datasets, ODI total score and PROMIS-29 Pain Interference, PROMIS-29 Fatigue and Sleep Disturbance, PROMIS-29 Anxiety and Depression, and PROMIS-29 Social Roles and Physical Function were grouped together. In the wearable sub-study, many objective measures related to PROMIS-29 Physical Function and Social Roles were grouped closely with watch features such as daily step counts, daily walking and running distance, and total daily stand time. The PHQ-9 metrics were only collected as part of the sub-study and were grouped in the same group as the PCS features. The heart rate variability was also grouped with these psychological factors. The NRS and PGIC were highly correlated (correlation = -0.82), but they were grouped in opposite directions of the first principal component. This is due to the way the scoring is defined in both scales (high NRS values mean more pain and high PGIC means better improvement). In the main study, we also collected a satisfaction question that was targeted at understanding how satisfied the subject was with pain relief received from the SCS device. This satisfaction question also grouped closely with the NRS in the main study. The first 3 principal components (PC) account for 72.9% of the variance in the REALITY main study (PC1 = 53.4%, PC2 = 11.6%, and PC3 = 8.1%) and 66.9% of the variance in the REALITY wearable sub-study (PC1 = 40.5%, PC2 = 18.4%, and PC3 = 8.0%) (Fig. 1). Score plots for the PROMs data of the main study and wearable sub-study data are shown in Fig. 2. This figure confirms the overlap of subject’s datapoint distributions in the wearable sub study with the larger Reality main study.

### Modeling of pain and PGIC using objective and subjective measures

**Fig. 3:**
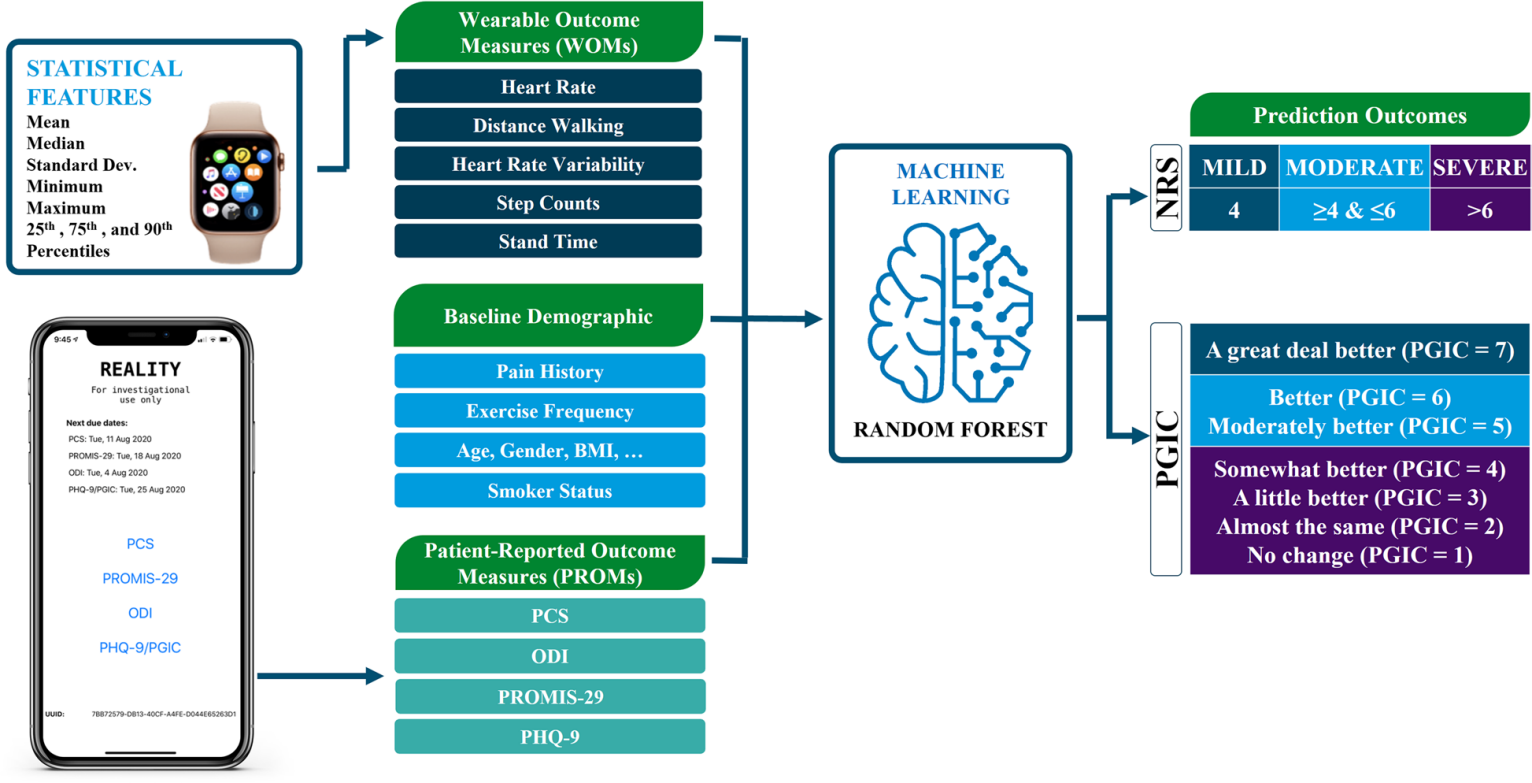
Data pipeline with different data sources (objective, subjective, and baseline demographics) used as an input to the machine learning model to predict pain and PGIC categories in the REALITY sub-study

The physical, physiological, and behavioral data collected actively and passively throughout the study using wearables (sub-study only) and questionnaires (both the main and sub-study) were used to construct machine learning models to classify PGIC and NRS in the study participants. A separate model for each of these two metrics was used. Figure 3 illustrates the flowchart for the prediction models including objective wearable data, subjective questionnaires, and baseline demographic and medical history as inputs for the sub-study. The main difference for the main study was lack of wearable data as an input to the models and PROMs collection happened in the clinic instead of on a custom application. Random Forest classification model was selected based on the superior performance and the ability to produce interpretable features compared with other machine learning techniques to predict both PGIC and NRS.

Table 2 summarizes the model evaluation metrics for predicting NRS and PGIC in both main study and the wearable sub-study. For the sub-study subjects, the subjective model for PGIC had the highest accuracy (Accuracy = 0.81 ± 0.06, F1 Score = 0.81 ± 0.07) compared to the wearable objective PGIC model that showed the accuracy of 0.75 ± 0.07 (F1 Score = 0.73 ± 0.08) (Table 2). The accuracy of PROMs NRS model was 0.81 ± 0.07 (F1 Score = 0.80 ± 0.07) and the NRS model for objective data had 0.78 ± 0.06 accuracy (F1 Score = 0.75 ± 0.07).

**Table 2:** Evaluation metrics and accuracies for the classification models

Study	Inputs	Output	Accuracy	F1 score	Specificity	Sensitivity
Wearable Sub-study	WOMs	PGIC	0.75 ± 0.07	0.73 ± 0.08	0.86 ± 0.04	0.69 ± 0.08
	PROMs	PGIC	0.81 ± 0.06	0.81 ± 0.07	0.91 ± 0.03	0.77 ± 0.07
	WOMs & PROMs	PGIC	0.82 ± 0.04	0.81 ± 0.05	0.91 ± 0.02	0.77 ± 0.05
	WOMs	NRS	0.78 ± 0.06	0.75 ± 0.07	0.83 ± 0.05	0.63 ± 0.10
	PROMs	NRS	0.81 ± 0.07	0.80 ± 0.07	0.87 ± 0.04	0.69 ± 0.11
	WOMs & PROMs	NRS	0.81 ± 0.06	0.80 ± 0.07	0.87 ± 0.05	0.68 ± 0.10
Main Study	PROMs	PGIC	0.71 ± 0.02	0.71 ± 0.03	0.82 ± 0.02	0.66 ± 0.03
	PROMs	NRS	0.64 ± 0.02	0.64 ± 0.03	0.81 ± 0.01	0.61 ± 0.02

**Fig. 4:**
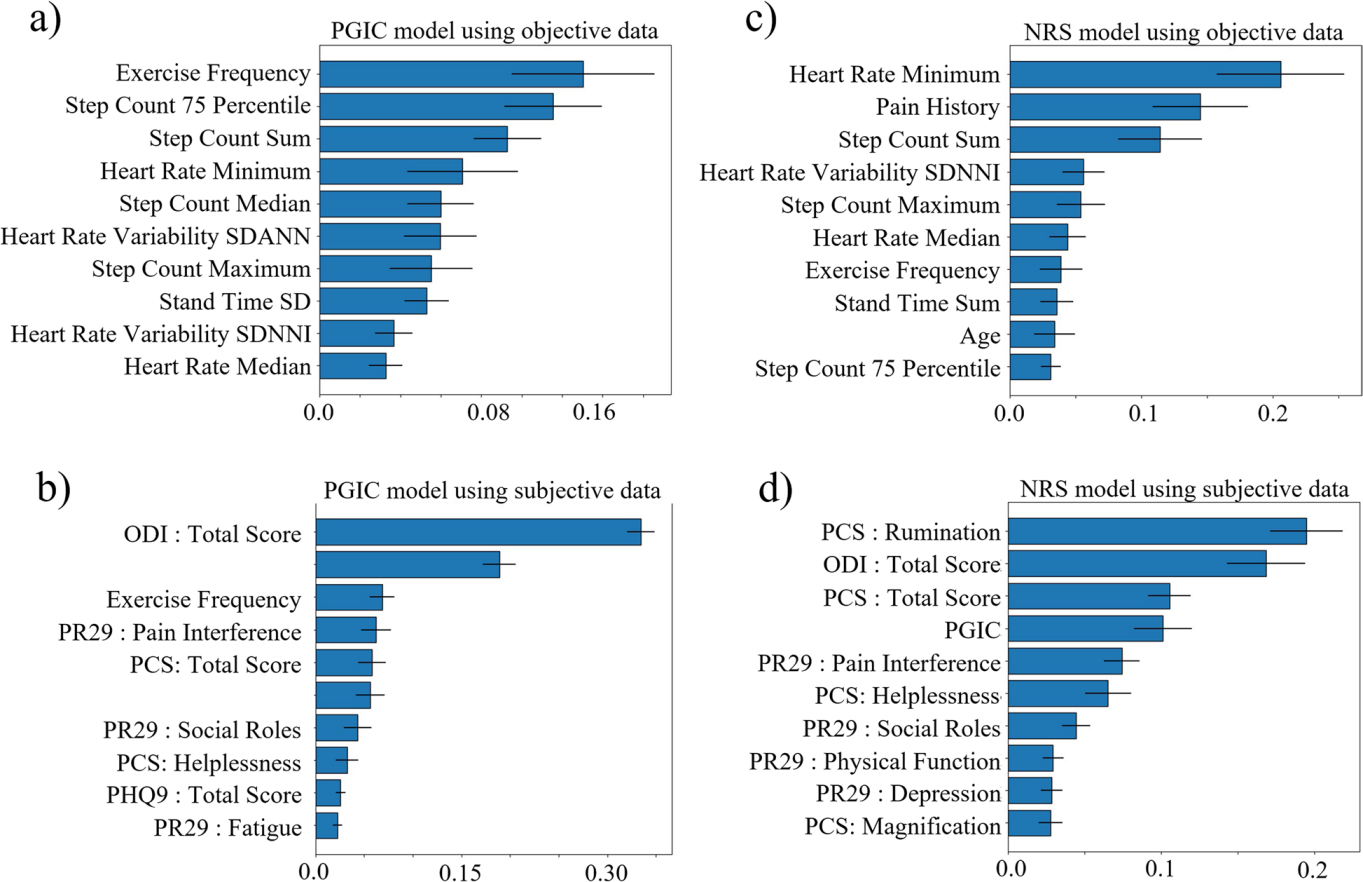
Average and standard deviation of feature importance for top 10 features in prediction models for REALITY wearable sub-study across 50 runs (**a**) PGIC prediction model using objective wearable measures as input; (**b**) PGIC model using subjective questionnaires plus baseline demographics as input; (**c**) NRS prediction model using objective wearable measures as input (**d**) NRS prediction model using subjective questionnaires plus baseline demographics as input. PR29 stands for PROMIS29 questionnaire

Figure 4 illustrates the average and standard deviation of the top 10 features across the 50 runs for prediction of the PGIC and NRS in the wearable sub study. For PGIC model using wearable measures (Fig. 4a), step counts, heart rate and heart rate variability post permanent implantation were among the top objective predictors, whereas PROMs such as, ODI total score, and pain average (NRS) were among the top subjective features (Fig. 4b). Baseline Exercise Frequency also showed as an important feature in both models for PGIC.

For NRS, heart rate, step counts, and heart rate variability were among the top objective features (Fig. 4c), whereas PROMs such as PCS rumination, ODI total score, PCS total score, and PGIC, were among the top subjective features (Fig. 4d). Baseline number of years patient is dealing with chronic pain (Pain History) also showed as an important feature in both models for PGIC. Supplementary Fig. A1, shows the average and standard deviation of top 10 input features for REALITY sub-study across 50 runs, using both objective wearable and subjective measures as inputs for predicting NRS and PGIC. Supplementary Fig. A2, illustrates the distribution of the top wearable input features over the 6-month period after the permanent implant for PGIC and NRS predictive WOMs models.

**Fig. 5:**
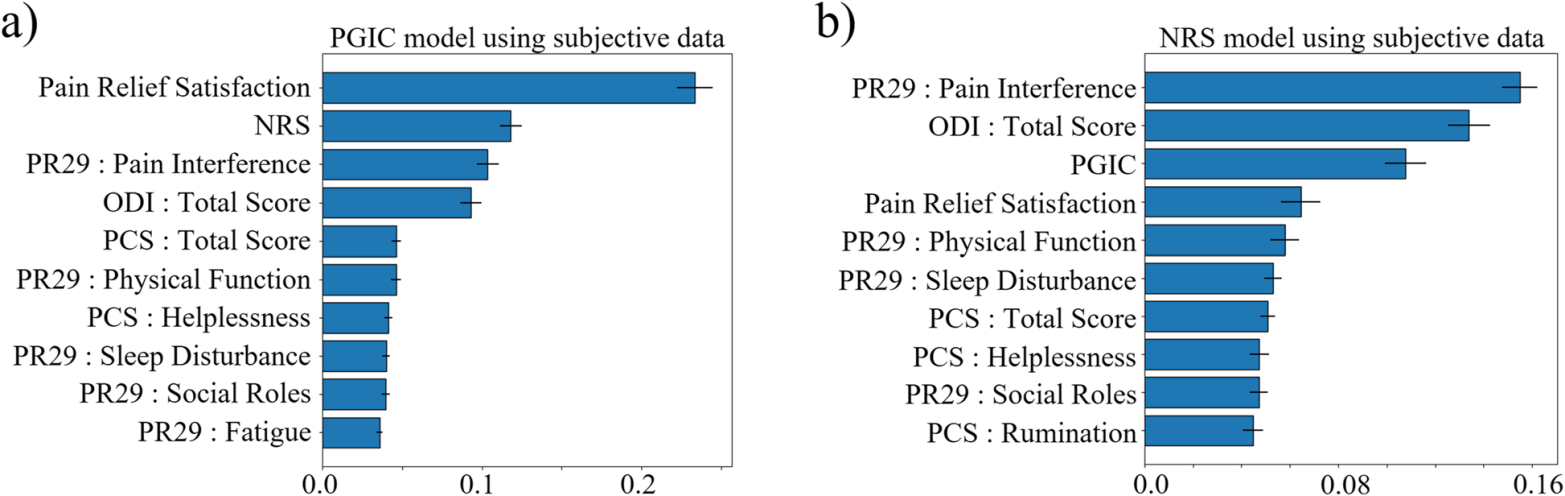
Average and standard deviation of feature importance for top 10 features in the PGIC (**a**) and NRS (**b**) prediction using subjective questionnaires, plus baseline demographics on REALITY main study. PR29 stands for PROMIS29 questionnaire

For the REALITY main subjects, only PROMs were available, and the model accuracy was lower compared to the sub-study model (F1 Score = 0.71 ± 0.03 and Accuracy = 0.71 ± 0.02). Figure 5a and b illustrates average and standard deviation of the top 10 features across the 50 runs for the prediction of PGIC and NRS using subjective measures in the REALITY main study. For both NRS and PGIC in the main study, PROMIS-29 Pain Interference, Social Roles and Activities, Sleep Disturbance, Fatigue, Physical Functions, and ODI total score were selected as the top features. The main difference between the two models’ top features was that the PGIC was an important feature for NRS perdition and vice versa the NRS was an important feature for PGIC prediction.

## Discussion

In this study, we utilized PCA dimensionality reduction to examine the similarities between objective and subjective measurements for chronic pain. We then applied machine learning techniques to predict SCS therapy outcome evaluated by subject’s response to NRS or PGIC using both subjective questionnaires and objective measures. The PCA dimensionality reduction results provided insights and confirmed the proximity of various patient-reported outcome and wearable objective measures in different clusters. The psychological aspects of pain, characterized with PCS subscales of helplessness, magnification, and rumination, were found to be closely associated with heart rate variability features from the wearable. The association between HRV and chronic pain has been previously observed (Telles et al. 2016; Goudman et al. 2021b; Koenig et al. 2016b). Our results confirm and expand on these findings in a more diverse chronic pain population treated with SCS. Not surprisingly, many movement-related wearable measures correlated with PROMs associated physical function, and social role participation. In addition, within the PROMs there were similarities between selected features such as Fatigue and Sleep Disturbance, Depression and Anxiety, and ODI and pain interference. This could allude to the fact that there are overlaps across questionnaires that can potentially be summarized to a shorter set of measures more suitable for frequent digital data collection. Prior research has emphasized the need for improved metrics to better characterize long-term patient response to neurostimulation therapy and overall subject satisfaction with the therapy though combination of global health measures and composite health scores (Hagedorn et al. 2022; Gewandter et al. 2021; Huygen et al. 2023; Pilitsis et al. 2021).

The NRS and PGIC were highly correlated in both studies. While the PGIC was originally developed as a measure of global change in health status, it has been adapted for use in a variety of specific conditions including chronic pain and has been used to assess therapeutic success in the absence of the NRS. Studies have shown that the PGIC can provide useful information about patients' pain experience, including changes in pain intensity, frequency, and impact on daily life (Suzuki et al. 2020). Our results showed that objective features were able to predict both PGIC and NRS outcomes with high accuracies even in the absence of subjective data in our sub study population.

Despite similarities of top features selected for predicting both NRS and PGIC, our model prediction accuracy was higher for PGIC compared with the NRS using subjective-only measures tested on the REALITY main study data with over 500 subjects. This was primarily driven by the top feature in the PGIC model, patient satisfaction. This feature had a higher correlation with the PGIC than the NRS as an indicator of response to therapy. Similarly, the PGIC questions, which queried activity limitations, symptoms, emotional state and quality of life, reflected an overall change since the study onset. This indicates that PGIC could be a better predictor of long-term therapy outcome as it allows patients to personalize and customize their response across numerous facets, including pain relief, sleep, and functional improvement over the period of treatment which are all of variable importance to different patient groups.

Subjective measures are crucial for evaluating physiological and psychological aspects of pain, but their frequent collection is burdensome for both patients and clinicians. In addition, subjective outcomes can be manipulated by patients, and third parties are often skeptical of their value in assessing outcomes. Wearable devices can objectively measure several features affected by pain, such as activity, sleep, psychological health, and social participation. The feasibility data from our wearable sub-study provides an objective measurement of many necessary biomarkers for continuous symptom monitoring, with minimal data loss (Patterson et al. 2023). The current study showed that objective features were able to predict both PGIC and NRS outcomes with high accuracy even in the absence of subjective data in our sub study population. Although the accuracy of our models in predicting NRS and PGIC are lower on the main study, the confidence bounds are tighter showing that models are more robust as they are trained on a larger population of patients.

Furthermore, it is important to compare the number of subject-specific data points in the two models developed on the main study and sub-study participants. In the main study, the model included 1100 datapoints from 557 unique subjects, or an average of 1.97 unique data points for each subject (in clinic visits). In contrast, the model included 142 rows of data from 15 subjects, or an average of 9.46 unique data points (through a custom digital application) for each participant. Although the number of subjects is fewer in the sub-study compared to the main study and the main study model could be a closer predictor of the general pain patient population, the sub-study model benefits from more frequent data points per patient that could help with better prediction of individual’s response over time. The lower number of data points per patient in the main study can also explain the reduced performance of the population model for predicting both NRS and PGIC compared to the sub-study. In addition, collecting data with a higher resolution through the digital application could increase the frequency of data collection for remote monitoring while reducing the burden of subjective data collection on people with chronic pain and clinicians. Developing personalized therapy is specifically beneficial given the variations across different patients and could potentially improve the individuals’ overall therapeutic effect and experience.

Our study had limitations as our findings are related to only one study that must be further tested on other studies. The small sample size used for developing machine learning models can affect the generalizability of our predictions across different patients. To mitigate this, we randomized the training and testing data 50 times and reported the average model performance. Additionally, the study lacked reliable sleep data, an important predicting factor for pain, due to inadequate time resolution and binary values provided for sleep data. While this study demonstrated feasibility in a small population of participants, future studies with larger sample sizes of people with chronic pain are necessary to address these limitations. Moreover, improvements in future generations of wearable devices can provide access to additional sensors and data, enhancing the robustness of predictive models for individual pain modeling.

This study provides a foundation for the development of digital biomarkers for pain using wearable devices and other digital technologies. By identifying the key physiological and psychological factors associated with pain, researchers can develop more accurate and precise digital biomarkers that can be used to monitor and treat pain in a more personalized and effective way. These biomarkers could be used to assess pain in real-time, monitor symptoms over time, and to inform clinical decision-making. We applied machine learning algorithms to a multi-dimensional data set consist of subjective and objective measurement to predict chronic pain patient response to SCS therapy assessed using two separate scales of NRS and PGIC.

These models could be beneficial to automate the monitoring of patient symptoms over time which enables remote monitoring of patients’ response to therapy and unlocks therapy for patients with remote geographical locations or limited access to specialized centers. Furthermore, these models can be used to detect adverse events and are the initial effort to develop a clinician-in-the-loop or a fully automated closed-loop system.

## Conclusions

This study analyzed data collected from a diverse population of patients with chronic pain to predict their response to SCS therapy using a combination of subjective and objective measures. The results suggest that PGIC can be a superior metric for predicting long-term outcomes and overall patient satisfaction compared to the NRS. Objective measures such as activity and heart rate measures obtained objectively from wearable devices were also found to be feasible in predicting therapy outcomes providing a foundation for developing digital biomarkers for pain. Although the study had limitations, it provides insights into the potential of using digital biomarkers to monitor and treat pain in a more personalized and effective way which could benefit individuals with limited access to healthcare. This work could bring us one step closer to a patient-centric digital health platform which could increase patient satisfaction with spinal cord stimulation therapy as well as the selection of suitable candidates to receive this therapy.

## Supplementary Information

**Additional file 1: Figure A1.** Average and standard deviation of feature importance for top 10 features in prediction models for REALITY sub-study across 50 runs, using both objective wearable and subjective measures as inputs for predictingPGIC, andNRS.

**Additional file 2: Figure A2.** Distribution of the top wearable input features over the 6-month period after the permanent implant for PGIC and NRS predictive WOMs models.

## Data Availability

The data will be available upon reasonable request or by the completion of the study according to the study protocol if a written request is made to and granted in writing by Abbott at Abbott’s sole discretion.
