# Supplementary figures and images for: Objective wearable measures and subjective questionnaires for predicting response to neurostimulation in people with chronic pain
Source: Bioelectron Med. 2023 Jun 21;9:13. doi: 10.1186/s42234-023-00115-4 (PMC10283222; doi:10.1186/s42234-023-00115-4)

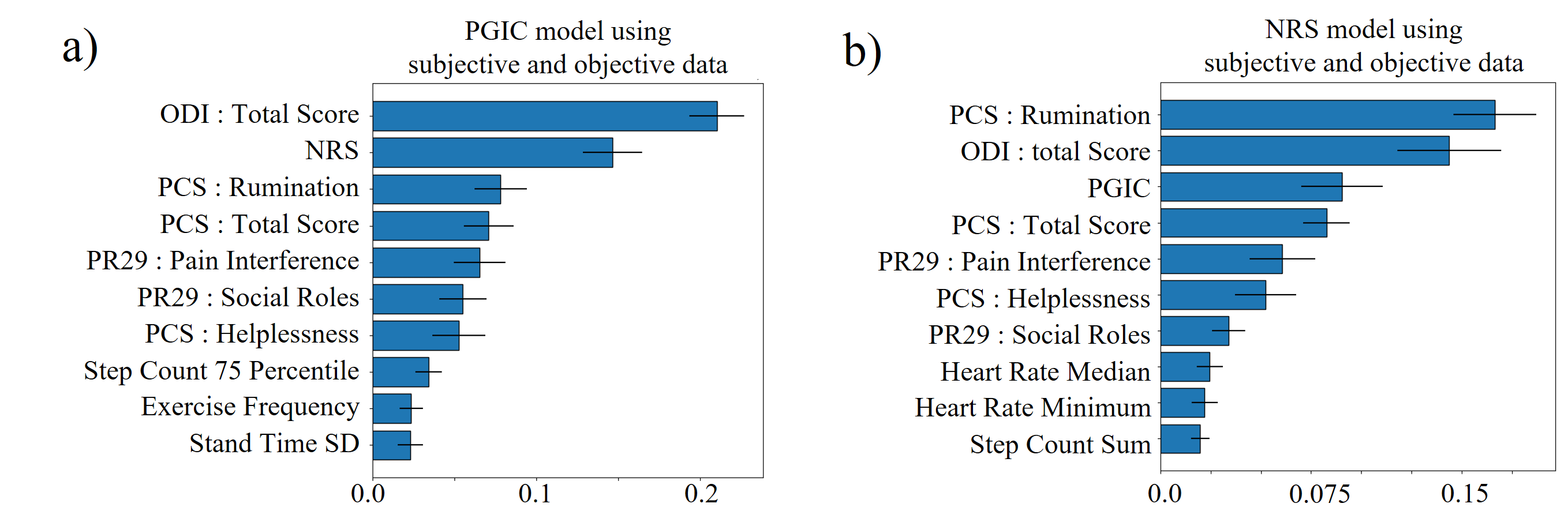

Supplement: Supplementary file 1 — Additional file 1: Figure A1. Average and standard deviation of feature importance for top 10 features in prediction models for REALITY sub-study across 50 runs, using both objective wearable and subjective measures as inputs for predictingPGIC, andNRS. [file 42234_2023_115_MOESM1_ESM.bmp]

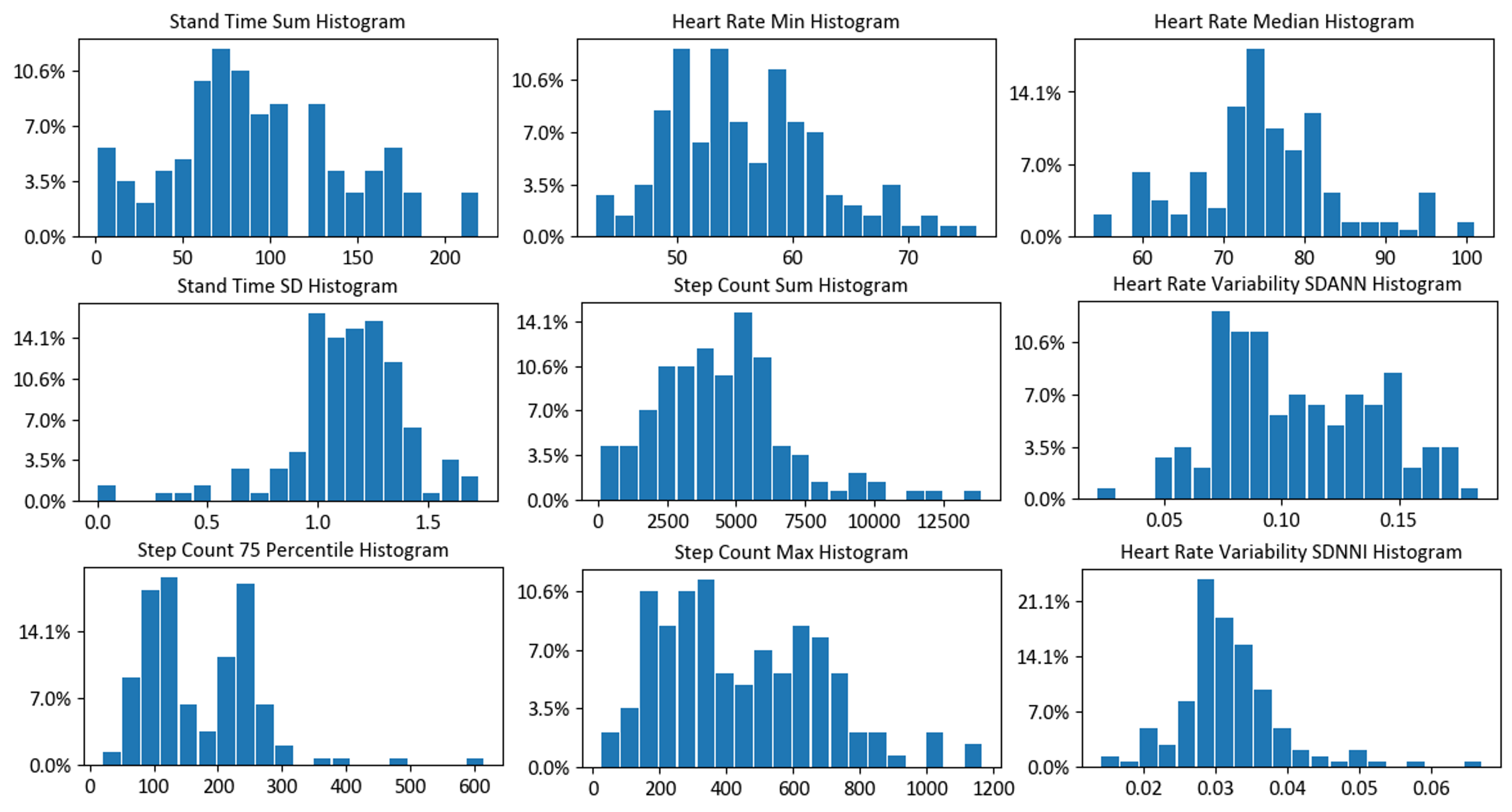

Supplement: Supplementary file 2 — Additional file 2: Figure A2. Distribution of the top wearable input features over the 6-month period after the permanent implant for PGIC and NRS predictive WOMs models. [file 42234_2023_115_MOESM2_ESM.png]
